# Characterization of the pathophysiological determinants of diarrheagenic *Escherichia coli* infection using a challenge model in healthy adults

**DOI:** 10.1038/s41598-021-85161-1

**Published:** 2021-03-15

**Authors:** Els van Hoffen, Annick Mercenier, Karine Vidal, Jalil Benyacoub, Joyce Schloesser, Alwine Kardinaal, Elly Lucas-van de Bos, Ingrid van Alen, Iris Roggero, Kim Duintjer, Anneke Berendts, Ruud Albers, Michiel Kleerebezem, Sandra ten Bruggencate

**Affiliations:** 1 Department of Nutrition and Health, NIZO, PO Box 20, 6710 BA Ede, the Netherlands; 2 Nestlé Institute of Health Sciences, Gastrointestinal Health, Nestlé Research, Lausanne, Switzerland; 3 Host Microbe Interactomics Group, Department of Animal Sciences, Wageningen University and Research, Wageningen, the Netherlands; 4 NutriLeads, Wageningen, The Netherlands; 5 Present Address: NutriLeads, Wageningen, The Netherlands

**Keywords:** Bacterial infection, Inflammation

## Abstract

An experimental human challenge model with an attenuated diarrheagenic *Escherichia coli (E. coli)* strain has been used in food intervention studies aimed to increase resistance to *E. coli* infection. This study was designed to refine and expand this challenge model. In a double-blind study, healthy male subjects were orally challenged with 1E10 or 5E10 colony-forming units (CFU) of *E. coli* strain E1392/75-2A. Three weeks later, subjects were rechallenged with 1E10 CFU of *E. coli*. Before and after both challenges, clinical symptoms and infection- and immune-related biomarkers were analyzed. Subset analysis was performed on clinically high- and low-responders. Regardless of inoculation dose, the first challenge induced clinical symptoms for 2–3 days. In blood, neutrophils, CRP, CXCL10, and CFA/II-specific IgG were induced, and in feces calprotectin and CFA/II-specific IgA. Despite clinical differences between high- and low-responders, infection and immune biomarkers did not differ. The first inoculation induced protection at the second challenge, with a minor clinical response, and no change in biomarkers. The refined study design resulted in a larger dynamic range of symptoms, and identification of biomarkers induced by a challenge with the attenuated *E. coli* strain E1392/75-2A, which is of value for future intervention studies. Addition of a second inoculation allows to study the protective response induced by a primary infection.

**Clinicaltrials.gov registration:** NCT02541695 (04/09/2015).

## Introduction

Diarrhea is an important cause of morbidity and mortality in all regions of the world and among all ages^1,2^. Enterotoxigenic *Escherichia coli* (ETEC) is one of the most common causes of infectious diarrhea and travelers’ diarrhea^2,3^. Although it is usually benign, travelers’ diarrhea represents a considerable socioeconomic burden for both the traveler and the host country^3^. Antibiotics can be a form of treatment, but the growing resistance of pathogens against antibiotics and potential side effects are a drawback. To date, an effective vaccine for ETEC infections is not available. Therefore, enhancement of resistance to food-borne infections by functional food ingredients is an attractive option.

The benefit of functional food ingredients in promoting resistance to infection can be studied in people who are travelling to a country of high risk for traveler’s diarrhea, or in other populations that are at risk for infections. An interesting alternative is to use controlled experimental infection challenge studies. Human ETEC challenge studies have played a major role in understanding disease pathogenesis and for testing the efficacy of investigational drugs and vaccines^4,5^. However, the ETEC strains used for these challenge studies often induce severe symptoms, requiring active intervention with antibiotics and oral and intravenous rehydration. For the development of functional food ingredients, these virulent strain models are considered less acceptable by ingredient manufacturers as well as ethical review boards. In contrast, the challenge model using a live-attenuated *E. coli* strain (E1392/75-2A) induces mild, self-limiting diarrhea, as well as mild gastrointestinal symptoms, which do not require antibiotic treatment^6–8^. The *E. coli* strain E1392/75-2A is a spontaneous mutant with deletion of the genes encoding the heat-labile (LT) and heat-stable (ST) toxins, whereas it still expresses an important colonization factor (Colonization factor antigen II, CFA/II). The exact mechanism by which this toxin-deficient strain elicits diarrhea is not known. The strain has been shown to confer 75% protection against re-infection with wild-type *E. coli* LT, ST, CFA/II strains, mediated by prevention of colonization of the proximal small intestine^9,10^. A well-characterized, relatively mild challenge model would be a valuable tool to help selecting functional ingredients targeting enteric infections.

Using this E1392/75-2A *E. coli* challenge model, beneficial effects of dietary interventions have been observed with dairy calcium-phosphate^6^ and dairy milk fat globular membrane^11^. So far, no effects have been observed with probiotics^7,8^.

At the standard inoculation dose, the short duration (1–2 days) and relative mildness of clinical symptoms limit the dynamic range in which effects of nutritional interventions can be assessed. Therefore, the present study was designed to refine the challenge model. The main objective was to increase the effect-size and/or duration of the clinical response to infection, and to further characterize gastrointestinal and immune factors underlying the clinical manifestations. To achieve that, a higher inoculum dose, as well as an adapted pre-challenge regimen was applied. Extensive biomarker analysis of blood and fecal samples was performed to obtain more insight into the kinetics of the host response to this infection and the relevance of these biomarkers. In addition, protection of subjects from diarrheal disease after exposure to a second challenge with the same strain was studied.

## Results

### Subject characteristics

Demographic and key baseline characteristics of the 42 healthy male volunteers who completed the study are presented in Table 1. Two subjects (both in the 1E10 group) did not continue in the study because of work and family-related circumstances (one subject dropped out before the first inoculation, one subject just after the first inoculation). These subjects were excluded from all analyses. No significant baseline differences were present between the study groups.

**Table 1:** Baseline and pre-challenge characteristics of study participants.

	Treatment groups		Subsets^c^	
	1E10 (n = 20)	5E10 (n = 22)	Low-responders (n = 12)	High-responders (n = 12)
**Baseline**^**a**^				
Age, year, median (range)^b^	21 (19–47)	21 (18–48)	21 (19–26)	21 (19–29)
Body mass index, kg/m^2^	23.3 ± 0.5	23.3 ± 0.5	22.1 ± 0.7	24.0 ± 0.6
Serum CFA/II-specific IgG, AU/mL, median (range)^b^	67 (13–427)	91 (20–364)	57 (13–427)	85 (20–245)
Serum total IgA, g/L	1.9 ± 0.1	1.8 ± 0.2	1.9 ± 0.2	1.8 ± 0.2
Fecal endogenous *E. coli*, × 10^3^ copies/mg feces, median (range)^b^	10 (0.1–2349)	37 (0.1–1979)	25 (0.1–2349)	45 (0.1–870)
Blood group A/AB/B/O	8/0/1/11	10/1/2/9	6/0/1/5	3/1/1/7
**Pre-challenge (day 12 or 13)**				
Fecal calcium, mg/day	477 ± 52	422 ± 42	449 ± 46	396 ± 70

^a^Data are expressed as mean ± SEM, unless stated otherwise.

^b^Subjects were stratified to the inoculation dose groups according to age, CFA/II-specific serum IgG titer at baseline, and fecal endogenous *E. coli* count.

^c^High- and low-responder subsets were defined by ranking all subjects based on a combination of all clinical outcomes after the first *E. coli* challenge (see “Methods” section). The sub-groups contained equal numbers of subjects from each dose group (n = 6 of each dose).

### Effect of inoculation doses on clinical symptoms upon the first and second *E. coli* challenge

**Figure 1:**
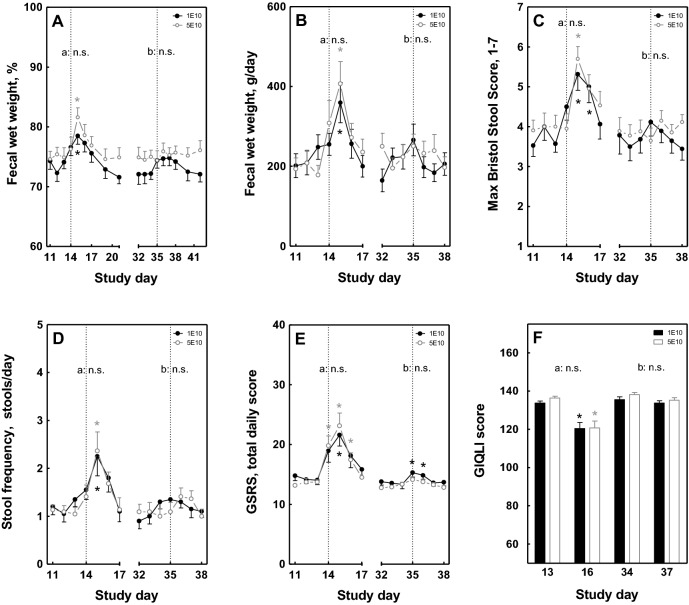
Effect of the dose of oral *E. coli* E1392/75-2A challenge on clinical parameters. Percentage and total fecal wet weight (**A**,**B**), stool consistency and stool frequency (**C**,**D**) and gastrointestinal disease-related symptoms and quality of life (**E**,**F**) were measured before and after infection with *E. coli* (strain E1392/75-2A) on study day 14 with standard dose 1E10 CFU (n = 20; black circles/bars) or high dose 5E10 CFU (n = 22; grey open circles/bars). On study day 35, all subjects received a second inoculation of 1E10 CFU of *E. coli* (n = 42). Results are expressed as means ± SEM. Timepoint at which significance is reached as compared to baseline day 13 (within the first infection period) or day 34 (within the second infection period) is indicated by *, with p < 0.05 within the specified group (repeated measures ANOVA ; black *: within standard dose; grey *: within high dose). Significant differences in the overall response between standard and high dose (two-way ANOVA) are indicated separately for the first (**a**, days 11–17) and second (**b**, days 32–38) infection period; *ns* not significant.

The *E. coli* challenge resulted in a similar significant increase in percentage and total fecal wet weight within the 1E10 and the 5E10 group, with a peak one day (day 15) after the first challenge, compared to pre-challenge (day 13) (Fig. 1A,B). Also the BSS and stool frequency showed a similar and significant increase after the first challenge (days 15 and 16) compared to pre-challenge within both dose groups (Fig. 1C,D). The kinetic pattern was very similar to the percentage and total fecal wet weight outcomes. On day 15, both groups reported a similar prevalence of WHO-defined diarrhea (39% of the 1E10 group and 40% of the 5E10 group).

Clinical symptoms, as scored by the GSRS, increased significantly within both the 1E10 and 5E10 group after the first challenge (days 14, 15 and/or 16) as compared to pre-challenge (Fig. 1E). The most prominent effects were observed in the GSRS subdomains diarrhea (loose stools, increased passage of stools and urgent defecation) and abdominal pain (abdominal pain and abdominal distention) (data not shown). For both dose groups, the first challenge was associated with a significant reduction in the GIQLI score as measured 2 days after the first challenge (day 16), as compared to pre-challenge (day 13) (Fig. 1F).

Upon the second *E. coli* challenge, a slight increase in the percentage fecal wet weight and total daily fecal wet weight, BSS and stool frequency was observed, mainly in the 1E10 group, but this was not significant (Fig. 1A–D). For the 1E10 group, the GSRS also showed an increase after the second *E. coli* challenge, which was mild but significant (Fig. 1E), whereas no effect on the GIQLI was observed (Fig. 1F). For all six parameters, no significant differences were observed in the response to *E. coli* between the 1E10 and the 5E10 group, both after the first and the second *E. coli* challenge.

### Clinical symptoms in high- versus low-responders

**Figure 2:**
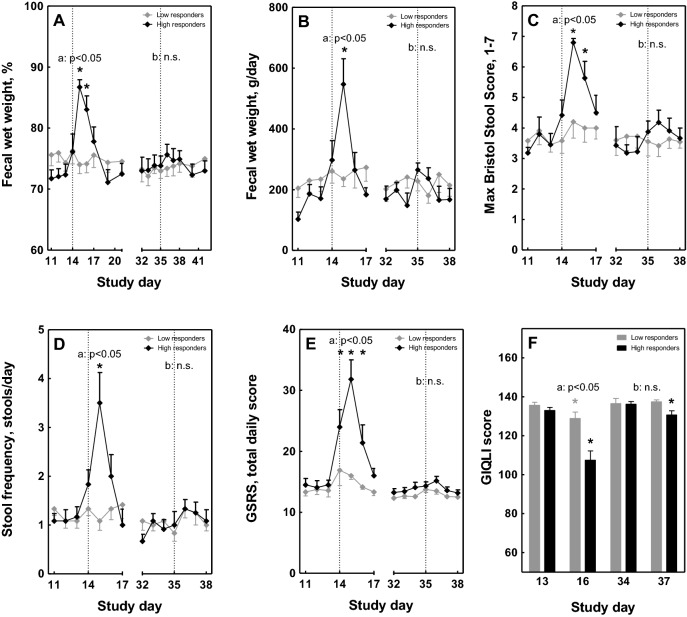
Effect of oral *E. coli* E1392/75-2A challenge in high- and low-responders on clinical parameters. Percentage and total fecal wet weight (**A**,**B**), stool consistency and stool frequency (**C**,**D**) and gastrointestinal disease-related symptoms and quality of life (**E**,**F**) were measured before and after infection with *E. coli* on study day 14 (strain E1392/75-2A; dose 1E10 CFU or 5E10 CFU). On study day 35, all subjects received a second inoculation of 1E10 CFU of *E. coli* (n = 42). Results are shown for a subset of high-responders (n = 12; black circles/bars) and low-responders (n = 12; grey diamonds/bars), selected based on ranking of a combination of clinical outcomes, and are expressed as means ± SEM. Timepoint at which significance is reached as compared to baseline day 13 (within the first infection period) or day 34 (within the second infection period) is indicated by *, with p < 0.05 within the specified group (repeated measures ANOVA; black *: within high-responders; grey *: within low-responders). Significant differences in the overall response between high- and low-responders (two-way ANOVA) are indicated separately for the first (**a**, day 11–17) and second (**b**, day 32–38) infection period; *ns* not significant.

Although no differences in clinical parameters were observed between the 1E10 and 5E10 dose, there was a clear diversity in the severity of the symptoms after the first *E. coli* challenge in both dose groups. Therefore, for further analysis of biomarkers, the 12 highest and 12 lowest responders were selected, based on a combination of the clinical scores (percentage and total fecal wet weight, BSS, stool frequency, GSRS and GIQLI). Figure 2 illustrates the analysis of all parameters between the high- and low-responders, confirming that the selection of these groups was correct. After the first *E. coli* challenge, a strong and significant increase in percentage fecal wet weight and total daily fecal wet weight, as well as in BSS and stool frequency, was only observed in high-responders (Fig. 2A–D). Also the GSRS symptom scores were strongly increased, whereas the GIQLI was strongly decreased in the high-responders (Fig. 2E,F). No or mild symptoms, and a slight decrease in the GIQLI, were observed in the low-responders after the first challenge (Fig. 2A–F). High-responders after the first *E. coli* challenge also showed a mild increase in percentage fecal wet weight and total daily fecal wet weight, as well as in BSS, stool frequency and GSRS upon the second *E. coli* challenge, but this effect was not significant. High-responders did report a significant decrease in the GIQLI after the second *E. coli* challenge, but this decrease in quality of life was less strong than after the first *E. coli* challenge. Low-responders did not have any change in clinical parameters after the second *E. coli* challenge (Fig. 2A–F).

### Reported adverse events

Adverse events related to the challenge, other than recorded by the GSRS, included headache (9.5% of subjects), fever (9.5% of subjects) and vomiting (7.1% of subjects). Frequency of these adverse events did not differ between dose or responder groups. No serious adverse events were reported during the study.

### Fecal *E. coli* E1392/75-2A

Fecal *E. coli* E1392/75-2A was detected in all subjects on the day after the first (day 15) and second *E. coli* challenge (day 36) in both the 1E10 and the 5E10 group (Supplementary Fig. 2). No significant differences were observed between the 1E10 and 5E10 group and between high- and low-responders. At day 49, *E. coli* numbers were near or below detection limit in all fecal samples.

### Fecal immune parameters in high- versus low-responders

**Figure 3:**
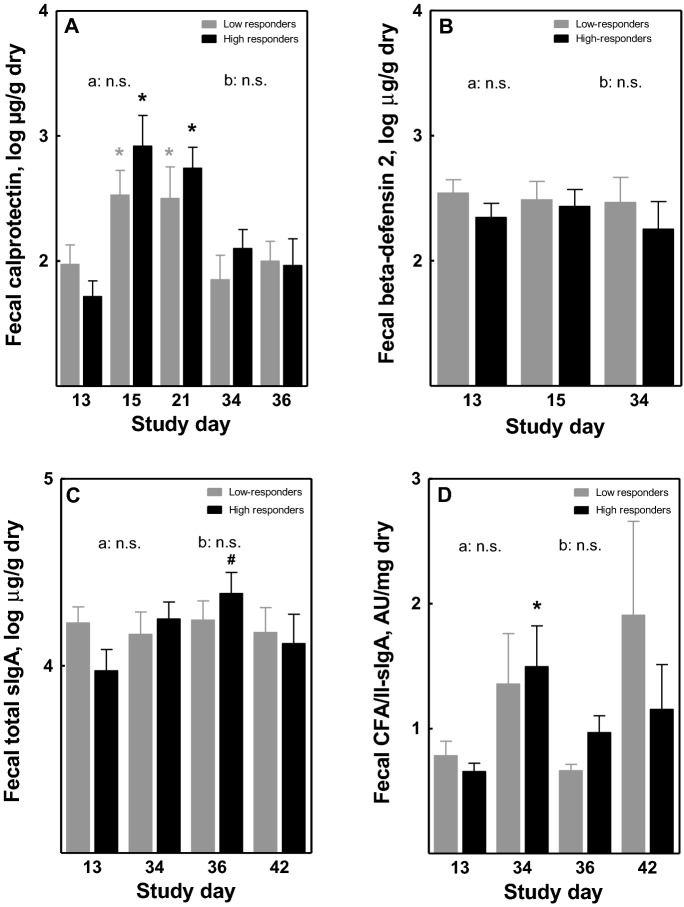
Effect of oral *E. coli* E1392/75-2A challenge on local fecal inflammatory and immune biomarkers. In fecal samples of high- and low-responders (n = 12 each), levels of calprotectin (**A**), β-defensin 2 (**B**), total sIgA (**C**) and fecal CFA/II-specific IgA (**D**) were analyzed. On day 14, subjects were orally infected with *E. coli* strain E1392/75-2A (dose 1E10 CFU or 5E10 CFU). On study day 35, all subjects received a second inoculation of 1E10 CFU of *E. coli*. Results are shown as means ± SEM. Timepoint at which significance is reached as compared to baseline day 13 (within the first infection period) or day 34 (within the second infection period) is indicated by *, with p < 0.05 within the specified group (repeated measures ANOVA; black *: within high-responders; grey *: within low-responders); ^#^p < 0.05 at day 36 compared to baseline day 13. Significant differences in the overall response between high- and low-responders (two-way ANOVA) are indicated separately for the first (**a**, days 13–34) and second (**b**, days 34–42) infection period; *ns* not significant.

To get insight into the mechanisms underlying the difference in clinical response between high- and low- responders, local immune parameters were analyzed in the high- and the low-responder subgroups. The *E. coli* challenge resulted in a significant increase in fecal calprotectin compared to pre-challenge (days 15 and 21 versus day 13), which was similar in both high- and low-responders (Fig. 3A). Fecal calprotectin returned to baseline levels before the second challenge, and no further increase was observed after the second challenge. The *E. coli* challenge did not affect fecal β-defensin levels (Fig. 3B). Fecal total sIgA was significantly increased two days after the second *E. coli* challenge (day 36) in the high-responders as compared to baseline (day 13), and declined on day 42, but was not affected by *E. coli* challenge in low-responders (Fig. 3C). The first *E. coli* challenge resulted in an increase in fecal CFA/II-specific IgA (day 34 versus day 13), which only reached significance in high-responders (Fig. 3D). The second challenge did not further increase fecal CFA/II-specific IgA. In contrast, levels seemed to decrease to baseline on day 36 after the second *E. coli* challenge and did not change significantly after the second challenge.

### Systemic inflammatory and immune parameters in high- versus low-responders

**Figure 4:**
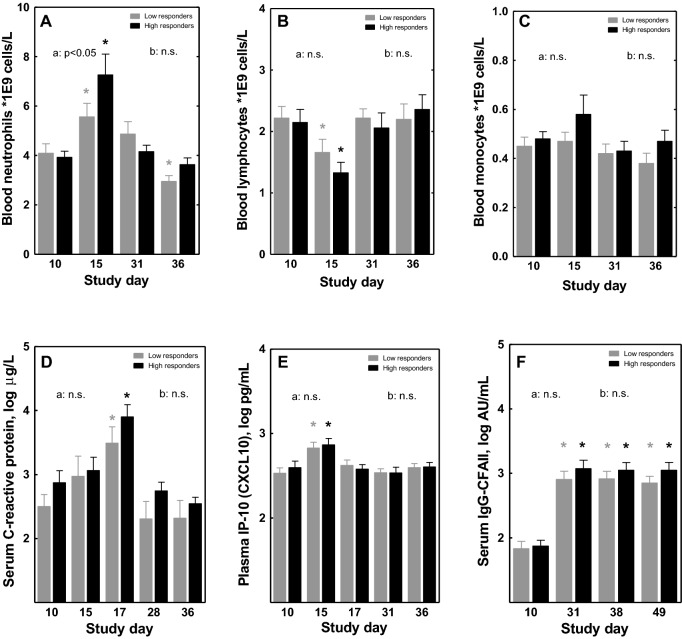
Effect of oral *E. coli* E1392/75-2A challenge on systemic inflammatory and immune biomarkers. In blood of high- and low-responders (n = 12 each), lymphocyte, neutrophil and monocyte counts were measured (**A**–**C**). In serum or plasma, levels of C-reactive protein, IP-10 (CXCL10) and CFA/II-specific IgG were measured (**D**–**F**). On day 14, subjects were orally infected with *E. coli* strain E1392/75-2A (dose 1E10 CFU or 5E10 CFU). On study day 35, all subjects received a second inoculation of 1E10 CFU of *E. coli*. Results are shown as means ± SEM. Timepoint at which significance is reached as compared to baseline day 10 (within the first infection period) or day 28/31 (within the second infection period) is indicated by *, with p < 0.05 within the specified group (repeated measures ANOVA; black *within high-responders; grey *within low-responders). Significant differences in the overall response between high- and low-responders (two-way ANOVA) are indicated separately for the first (**a**, days 10–31) and second (**b**, days 28/31–49) infection period; *ns* not significant.

**Figure 5:**
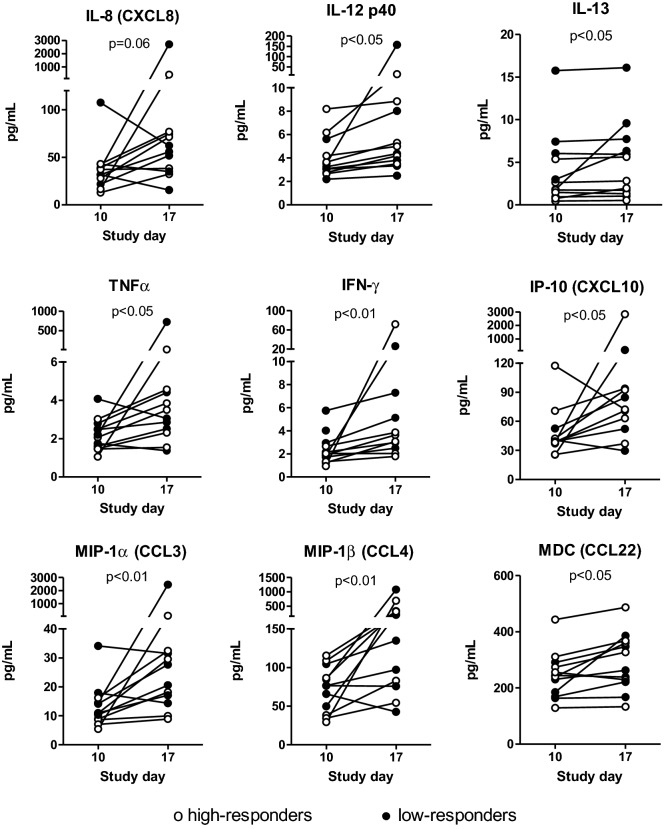
Ex vivo cytokine and chemokine production in whole blood before and after *E. coli* challenge. Ex vivo stimulation of whole blood cells was performed in a subset of subjects (n = 6 low-responders, closed circles, and n = 6 high-responders, open circles). Subjects were orally infected with a live attenuated *E. coli* strain E1392/75-2A on study day 14. Whole blood stimulation was performed on study day 10 and 17, using Null TruCulture tubes (medium only). No difference was observed between low- and high-responders, therefore the data of both subgroups were combined in statistical analysis. From the total panel of cytokines and chemokines that were analysed, the figures are shown for those that changed (borderline) significantly after challenge (Wilcoxon Signed Rank test).

As markers of systemic inflammation, both the high- and low-responders showed a similar and significant increase in neutrophils and a decrease in lymphocytes (day 15, Fig. 4A,B) after the first challenge, but no change in monocytes (Fig. 4C), basophils, eosinophils, erythrocytes or thrombocytes (data not shown). The neutrophil count, although increased, remained within the normal cutoff values. Serum C-reactive protein (CRP, measured by high-sensitive ELISA) (day 17, Fig. 4D), and plasma IP-10 (CXCL10, measured by Luminex) (day 15, Fig. 4E) also showed a similar and significant increase in high- and low-responders after the first challenge. All these inflammatory parameters returned to baseline before the second challenge, and were not affected by the second challenge. Other cytokines measured in plasma (IFN-γ, IL-1β, IL-6, IL-8 (CXCL8), TNF-α and MIP-1α (CCL3)) were below detection limits. As a marker of adaptive immune activation, CFA/II-specific serum IgG increased significantly from baseline in both high- and low-responders on day 31 and stayed elevated until day 49 (Fig. 4F). Production of cytokines/chemokines was also analyzed in supernatant from ex vivo TruCulture null- and LPS-stimulated whole blood in a subset of subjects (n = 6 high-responders and n = 6 low-responders) before and after the first challenge (Fig. 5, Supplementary Fig. 4, Supplementary Tables 1A,B). No differences were observed in the response between high- and low- responders. Therefore, the data of these subgroups were combined in further statistical analysis. Several cytokines and chemokines increased after challenge in the unstimulated cells (“null” condition), namely IL-8 (CXCL8) (trend p = 0.06), IL-12p40, IL-13, TNF-α, IFN-γ, IP-10 (CXCL10), MIP-1α (CCL3), MIP-1β (CCL4) and MDC (CCL22) (all p < 0.05), although the effect size for some was small (i.e. IL-12p40, IL-13, IFN-γ, TNF-α). All other cytokines and chemokines assessed remained unchanged (Supplementary Table 1A). In the LPS-stimulated condition, many cytokines and chemokines were strongly induced, and most of them remained unchanged after challenge (Supplementary Table 1B). A significant increase was observed for IL-1β, IL-2, IL-6, IL-12p40, and IL-15, whereas a significant decrease was observed for IL-8 after the first challenge (Supplementary Fig. 4). With the exception of the effect for IL-8, the effect size for the other cytokines was small, despite being significant.

## Discussion

The *E. coli* infection challenge applied in this study, has been used previously to assess the impact of nutritional interventions on resistance against bacterial gut infection. This approach is in line with regulatory requirements to demonstrate significant impact of a food ingredient on prevalence, severity or duration of clinical symptoms in support of a health claim for such a food ingredient^12^. One of the potential limitations of the model was the short duration (1–2 days) and the relative mildness of the clinical symptoms at the standard inoculation dose of 1E10 CFU, which limits the dynamic range to detect significant effects in nutritional intervention studies. Furthermore, although several biomarkers have been shown to be associated with the response to infection^6–8,11^, the kinetic responses of these biomarkers have not been studied in detail. Therefore, the current study had three goals: first, to achieve a higher effect-size by using a higher inoculation dose of the *E. coli* E1392/75-2A strain, introduction of a standardized low-calcium evening meal prior to challenge, an extended duration of fasting prior to the challenge, and restricted intake of specific mediation and alcohol; second, to obtain more insight into the kinetics of the host response to this infection by extensive biomarker analysis in blood and fecal samples; third, to determine if exposure to a first challenge protects volunteers against re-challenge with the same strain.

The diarrhoeagenic *E. coli* strain E1392/75-2A used in this study is a spontaneous mutant in which the genes encoding the LT and ST toxins are deleted. Consequently, this strain does not produce any toxins, but still expresses CFA/II^9,10^. Since toxin production is considered critical for diarrhea induction by *E. coli*, it is remarkable that this toxin-deficient strain still induces diarrhea and gastrointestinal symptoms^6–8,11^.

In order to increase the dynamic range regarding severity or duration of symptoms in this *E. coli* challenge model, the oral inoculation dose of *E. coli* was increased from 1E10 to 5E10. The study shows that the duration as well as the severity of symptoms to the first inoculation of 5E10 CFU were similar to the 1E10 CFU inoculate. Symptoms were mild, and all recorded disease episodes were self-limiting and did not require antibiotic treatment. For *E. coli* strain E1392/75-2A, no dose–response studies have been published. A dose of 2E10 CFU has been used to evaluate the heterologous protective immune response induced by this strain towards a secondary infection with a wild-type strain^9^. The primary infection in that study induced mild diarrhea in 15% of the subjects. However, no dietary restriction regarding calcium were applied. With subjects on a calcium-restricted diet, the regular dose of 1E10 CFU of this strain, as used in the current study, was already shown to still give relatively mild symptoms, but in a larger proportion of subjects^6–8,11^. The exact dose at which the maximum response is reached may depend on the type of *E. coli* strain used^5^. Most strains that have been tested in other studies are wild-type toxin-producing strains, with a dose ranging from 1E6 to a maximum of 1E10 CFU. For some wild-type strains, a five-fold increase of the dose was shown to impact on the severity of diarrhea^5^. Therefore, a five-fold higher dose of 5E10 CFU as compared to the standard dose of 1E10 was selected as high dose in the current study. The similar outcomes for both inoculation dosages suggest that either the clinical response to the 1E10 CFU inoculate of the *E. coli* E1392/75-2A strain is the maximum effect-size, or that a fivefold increase in inoculation dosage was not enough to achieve a higher effect-size.

The severity of the symptoms, both for the 5E10 and the 1E10 CFU dose, was similar to previous studies, in which 1E10 has been used^6–8,11^. Interestingly, the duration of clinical symptoms was prolonged in the present study to 2–3 days, compared to 1–2 days observed in the previous studies. As this was independent of the dose, this is likely due to refining other aspects of the study design, such as extended fasting (from 20:00 pm the previous day rather than from 4 h before infection), the introduction of a standardized low-calcium meal prior to the inoculation day, or restrictions on alcohol and medication intake on the 7 days around the infection (both not included in previous studies). The extended duration of clinical symptoms accounted for all reported parameters (stool consistency, stool frequency and GSRS), with a similar time window and pattern of the kinetic response. This pattern also corresponded well to the more objective parameters percentage and total fecal wet weight.

In addition to the clinical symptoms, the levels of local and systemic innate immune parameters were increased after *E. coli* challenge, i.e. decreased blood lymphocytes, and increased blood neutrophils, serum CRP, and fecal calprotectin. The data show that this is a rapid response induced directly after infection, which declines after 2–3 days. Similar to the clinical symptoms, infection dose had no impact on the effect size for these parameters. For fecal calprotectin and serum CRP, their increase is in line with results from previous *E. coli* challenge studies with the same E1392/75-2A strain^8,11^. Ex vivo non-stimulated whole blood cells also showed a significant increase in several cytokines and chemokines after *E. coli* challenge, reflecting a systemic activation of immune functions. This systemic activation was confirmed by the observed increase of CXCL10 in plasma, and by the increase in neutrophils (probably recruited from the bone marrow)^13^ and the decrease in lymphocytes (probably homing to the lymph nodes in the gut)^14^. Neutrophils and CXCL10 are known to be induced upon bacterial and viral infection^15–17^. CXCL10 can be produced by a variety of cells, including neutrophils, in response to IFN-γ and LPS^18^. To our knowledge, this is the first *E. coli* infection challenge study confirming the effect of infection on these parameters.

Together, these data indicate that both local and systemic key inflammatory features of acute GI infection are detected in this *E. coli* challenge model, which can serve as biomarkers to follow the innate immune response to *E. coli* infection.

In the present study, a second infection challenge was included to acquire more insight into the protective response to re-infection induced by the primary challenge. This study clearly demonstrated that subjects in both dose groups were largely protected upon exposure to an additional challenge with the same strain, as hardly any clinical response was observed to the second inoculation. Homologous protection was also seen previously in studies using a challenge with wild-type *E. coli* strains^19^. This protection may be mediated by fecal antibodies to *E. coli*, that may prevent adhesion of the bacteria to the gut epithelium. Fecal CFA/II-specific IgA was indeed increased after the first inoculation. Specific IgA levels tended to temporarily decrease early after the second challenge (day 36), which may be due to binding to the *E. coli* bacteria. Levels of CFA/II-specific serum IgG also strongly increased after the first inoculation, which may have contributed to the protection against the second challenge. The levels of CFA/II-specific fecal IgA and serum IgG did not further increase after the second inoculation, which may suggest that the levels reached their maximum upon the first inoculation.

In addition to the clinical response, also local and systemic innate and adaptive immune parameters were not impacted after the second challenge. For the CFA/II-specific serum antibody response, this is in contrast to an earlier study in which a re-challenge did elicit a further increase in the serological response to CFA/I^19^. This discrepancy might be explained by difference in the *E. coli* strain used, the primary inoculation dose (wild-type H10407 at 1E8 CFU versus E1392/75-2A at 1E10 and 5E10 CFU in our study), or the length of time between the 2 challenges (2–3 months versus 3 weeks in our study). Another explanation may be the fact that the attenuated *E. coli* results in less severe diarrhea than the wild-type strain, thereby allowing better mucosal pathogen adherence and intestinal colonization, resulting in a better immune response already at the first challenge.

Surprisingly, protection was not observed microbiologically, since the detected *E. coli* count in fecal samples of subjects after re-challenge was similar to that found after the first challenge. However, fecal *E. coli* counts of infected participants might not necessarily reflect small intestinal colonization, but rather survival and multiplication of *E. coli* in the large intestine^20^. After causing acute diarrhea followed by clinical recovery, some enteropathogens, including *E.* coli^8,11,21^ and the strain used in this study, can be excreted asymptomatically for days to weeks after infection. This suggests that the lack of symptoms upon re-challenge is not due to absence of detectable *E. coli* counts, but more likely to prevention of *E. coli* adhesion in the small intestine, as described above.

Interestingly, a subgroup of the participants displayed very low responses to the primary challenge in terms of clinical outcomes. Asymptomatic subjects are common in human challenge studies with wild-type ETEC strains^5,21^. They were also observed in previous studies with the attenuated *E. coli* strain used in this study (unpublished observations). This indicates that some host factors might be involved in the predisposition to develop diarrheal illness following ingestion of a pathogen. Remarkably, most of the biomarkers measured in high- and low-responders seemed to be induced at equal levels, irrespective of the difference in the magnitude of the clinical response after infection. This suggests that the local and systemic inflammatory and immune markers that were measured in the present study are not causally related to the immediate clinical response to infection, but reflect the response to exposure to a high dose of pathogen irrespective of clinical symptoms.

As a first step during infection, *E. coli* adheres to specific receptors on the surface of human intestinal epithelial cells, which includes sugar moieties of glycoproteins or mucins as well as surface proteins, such as glycosphingolipid receptors asialo-GM1 and GM2^22,23^. Susceptibility to infection may be affected by the presence or absence of these receptors, or the expression of variant receptors^21^. Glycosphingolipid receptors have been shown to be upregulated during RSV infection, which is important for control of RSV infection^24^. Whether this upregulation also occurs upon *E. coli* infection is not known. It has been suggested that blood group may impact on the response to infection, with blood group A or AB, or Lewis(a + b-) non-secretors having a higher frequency or being more sensitive to CFA/I-expressing *E. coli* infection^25,26^. The potential role of ABO blood group in high and low responders was also determined in the current study (Table 1). Blood group A or AB occurred with similar frequency in high and low responders (4/12 versus 6/12, not significant), suggesting that blood group did not affect the severity of the response to the attenuated *E. coli* strain, at least in this Dutch study population. For future studies, better understanding of the factors that determine the clinical response to infection would be valuable, to allow selection of a more homogeneous study population for nutritional intervention studies.

Comparison of various *E. coli* strains in in vitro experiments confirmed that *E. coli* E1392/75-2A display very similar binding capacity to intestinal epithelial cells, as well as induction of IL-8 production in intestinal epithelial cells, as compared to closely related (H10407) as well as more distant (B7A and E243744A) toxin-producing *E. coli* strains (Supplementary Fig. 3A,B). However, unlike these toxin-producing strains, the *E. coli* E1392/75-2A strain fails to elicit a rapid disruption of the epithelial barrier in a TEER assay (Supplementary Fig. 3C,D). Taken together with the results from the current clinical study, these data indicate that the clinical symptoms elicited by *E. coli* E1392/75-2A are independent of toxin-induced loss of mucosal integrity, and likely depend on the bacterial attachment to the intestinal mucosa and the subsequent induction of pro-inflammatory mediators, implicating the inflammatory responses of the host as one of the factors involved in the observed clinical symptoms.

The experimental *E. coli* E1392/75-2A challenge model is a valuable tool to evaluate the efficacy of food interventions to protect against diarrhea before moving on to field trials^5–8,11^. However, an important limitation of these type of challenges is the fact that, for ethical reasons, they can only be performed in adults. This does not fully represent the population with the highest disease burden, namely young children living in ETEC endemic regions^20^. Interestingly, the toxin-deficient *E. coli* E1392/75-2A strain used in this study has been shown to confer 75% protection against re-infection with wild-type, toxin-producing CFA/II *E. coli* strains^9^, making this strain a relevant model strain to use in experimental challenge studies. However, as toxins are lacking in this strain, it is obviously not a suitable model to study toxin neutralization effects of food ingredients.

In conclusion, the current study design resulted in an extension of clinical symptom duration as compared to previous studies, and thereby provides an expanded window of measurement for the potential benefits of nutritional interventions. In addition, an enlarged set of local and systemic innate and adaptive immune biomarkers was identified which could support understanding of the mode of action the nutritional interventions. The addition of the second inoculation could provide an opportunity to study the protective response induced by a primary infection. Identification of baseline markers allowing to predict clinical responsiveness of participants would strongly benefit this challenge model but deserves further studies.

## Methods

### Study design and ethics

The study was a randomized, double-blind, parallel infection trial conducted in healthy male adults in the clinical unit of NIZO, the Netherlands (Supplementary Fig. 1), during the first quarter of 2016. The study was approved by the Medical Ethics Committee of Wageningen University, the Netherlands, and registered at clinicaltrials.gov (Identifier: NCT02541695). Written informed consent was obtained from all study participants before screening for eligibility. The study was conducted according to the principles of the Declaration of Helsinki (Fortaleza, Brazil, 2013) and according to the Dutch Medical Research involving Human Subjects Act.

### Study participants

**Figure 6:**
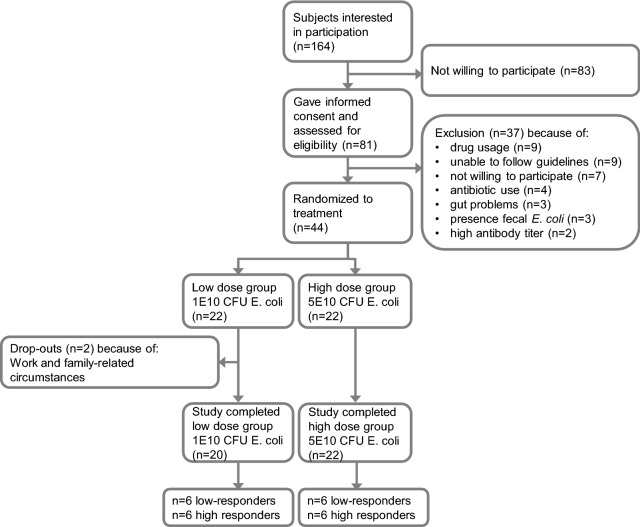
Flow diagram of study subjects.

Healthy male volunteers, 18–55 years of age, were recruited in November and December 2015. Women were not eligible for this study, because of the disadvantage of the female anatomy hindering fecal sample collection without urine contamination during acute infection, as well as interference of abdominal symptoms during their menstrual cycle. Forty four eligible healthy male volunteers who fulfilled all the criteria were selected to participate in the present study (Fig. 6). The exclusion criteria were: current or previous underlying gastro-intestinal disease; vaccination for or ingestion of ETEC, cholera, or *E. coli* heat labile toxin within 3 years prior to inclusion; known allergy to antibiotics; reported average stool frequency of < 1 or > 3 per day; symptoms consistent with travelers' diarrhea within 3 years prior to inclusion; use of antibiotics, norit, laxatives up till 6 months prior to inclusion; use of gastric acid suppression medication within 3 months prior to inclusion; current excessive alcohol consumption or drug (ab)use; vegans. In addition, subjects with high IgG antibody titers against CFA/II, IgA deficiency, or detectable fecal *E. coli* E1392/75-2A counts at screening, were excluded from participation in the study because of likely altered resistance to the *E. coli* strain administered in the present study^10^. For screening, blood was collected and analyzed for specific serum IgG against *E. coli* E1392/75-2A CFA/II, as described previously^6,7^, and total IgA was determined using a routine diagnostic immunoturbidimetric assay. In addition, a fresh fecal sample was obtained and analyzed for presence of *E. coli* E1392/75-2A by qPCR, as described before^8^.

### Study guidelines and compliance

Subjects were instructed to maintain their habitual pattern of physical activity and food intake. As dietary calcium was shown to increase resistance to *E. coli*-challenge in a previous human trial, they were instructed to limit their dietary calcium intake to on average 500 mg/day, by excluding dairy products from their diet, starting two weeks before *E. coli* challenge, and during the whole study period^6^. Dairy products were replaced by providing participants with low-calcium soy deserts (Bio Soya Desert Provamel, Alpro Soja Nederland BV, Breda, The Netherlands). To verify whether subjects adhered to the low-calcium dietary restrictions, fecal calcium was analyzed in homogenized fecal samples collected before *E. coli* challenge (on study days 12 or 13), as described previously^6^.

The use of non-steroidal anti-inflammatory drugs, gastric acid suppression or anti-motility medication, and antibiotics was prohibited on the three days before, during and 3 days after the diarrheagenic *E. coli* challenge, unless needed as rescue medication. Paracetamol up to 2 g/day was allowed as rescue medication during these days. Adherence to study guidelines was recorded in an online subject diary.

### Group size, randomization and stratification

Sample size (n = 22 subjects per group) was based on percentage fecal dry weight, as reported in a previous study^6^, with two extra subjects per group to compensate for potential dropouts. A change in percentage of fecal dry weight from 25 to 21% was considered a relevant effect. Based on two-sided statistical testing for unpaired data, α = 0.05 (chance on type-I error) and β = 0.20 (chance on type-II error), it was calculated using the software package Statistica (2015) that 20 subjects per group were required (µ1 = 25.1; µ2 = 21.0; SD = 4.3, α = 0.05, β = 0.20).

Subjects were stratified according to age, CFA/II-specific serum IgG titer, and endogenous fecal *E. coli*, and were randomly assigned to the high (5E10 CFU) or standard-dose (1E10 CFU) infection group. Stratification and randomization (using a random number generator) of study subjects and labelling of the *E. coli* doses was performed by a non-blinded person not involved in the study. All researchers of the project team, as well as the study subjects, were kept blind to assignment of treatment. The randomization code of each subject was broken after finishing laboratory and data analyses of the primary and secondary outcomes.

### Oral diarrheagenic *Escherichia coli* challenge

On day 13, subjects consumed a standardized low-calcium evening meal, and fasted from 20.00 PM overnight until the *E. coli* challenge. On day 14, subjects received a single oral dose of the attenuated diarrheagenic *E. coli* strain E1392/75-2A (standard dose 1E10 or high dose 5E10 CFU). Under supervision, subjects first drank 100 mL of demineralized water containing 2 g sodium bicarbonate (Merck, Darmstadt, Germany) to neutralize the gastric acid. After 5 min, subjects drank 100 mL of diluted fruit juice, set at a pH of 7.4 containing the *E. coli*. Subjects were not allowed to eat or drink for 1 h after challenge. At day 35, all subjects underwent a second challenge, consisting of a single oral dose of 1E10 CFU of the same *E. coli* strain, which was performed according to the same procedure. The period of 3 weeks between first challenge and rechallenge was based on previous experience, showing that symptoms induced by this strain are of short duration, and the challenge strain was largely cleared within that period. The aim was not to achieve optimal antibody response, but to obtain a secondary clinical or immune response that might be modifiable by food ingredients, as an extension of the prime challenge model.

### Infectious diarrhea, stool consistency, stool frequency, symptoms and quality of life

Three days before, the day of and 3 days after each *E. coli* challenge (study days 11–17 and 32–38), 24 h complete fecal samples were collected. On study days 19, 21, 28, 40, 42 and 49, spot fecal samples were collected. Fecal samples were frozen at − 20 °C immediately after defecation at the subject’s home. Frozen samples were transported to the lab, weighed, homogenized, and aliquots were stored at − 20 °C for later analyses. Response to infection was measured by daily total fecal wet weight excretion (for 24 h fecal samples, by weighing all stool samples collected between 7 a.m. in the morning until 7 a.m. the next day), and by the percentage fecal wet weight (in 24 h fecal samples as well as in spot fecal samples) as determined by freeze-drying. Percentage wet weight is an objective measure of stool consistency, discriminating between loose and well-formed stools.

On study days 11–17 and 32–38, subjects also reported daily information on stool consistency by using the Bristol Stool Scale (BSS)^27^, stool frequency and gastrointestinal symptoms according to the validated Gastrointestinal Symptom Rating Scale (GSRS)^28^ in an online subject diary. The GSRS is a disease-specific instrument of 15 items combined into 5 symptom clusters: reflux, abdominal pain, indigestion, diarrhea, and constipation. For the present study, the symptom cluster reflux was not recorded. Diarrheal episodes were defined according to WHO criteria, as passage of three or more loose or liquid stools per day^29^, based on reported BSS scores.

Subjects reported their quality of life over the past three days, on the day before the first and second infection challenge (study days 13 and 34) and two days after both infection challenges (study days 16 and 37), using the Gastrointestinal Quality of Life Index (GIQLI). The GIQLI is a gastrointestinal disease-specific quality of life instrument with 36-items^30^, with a total score ranging from 0 to 144. Higher scores represent better health-related QL.

### Fecal endogenous *E. coli* and *E. coli* strain E1392/75-2A

In fecal samples before and after each *E. coli* challenge, presence of *E. coli* strain E1392/75-2A using Colonization Surface antigen 3 (CS3)-specific primers, and total fecal endogenous *E. coli*, was quantified by qPCR amplification using a Bio-Rad CFX384 Real-Time System (C1000-Thermal Cycles) with Bio-Rad methodology (iTaq Universal Probes Supermix; Bio-Rad), as described previously^8,31^.

### Intestinal and systemic immune parameters

Before and after each *E. coli* challenge, fecal calprotectin, human β-defensin 2 (HBD-2) and total secretory IgA were measured using a commercially available kit (Immundiagnostik AG, Bensheim, Germany) according to manufacturer’s instructions.

Fecal CFA/II-specific IgA and serum CFA/II-specific IgG were measured by home-made ELISA. ELISA plates were coated overnight at 4 °C with CFA/II antigen (2.5 μg/mL in PBS). Plates were washed with wash buffer (PBS/0.05% Tween20), and (for fecal IgA) incubated with 200μL PBS/4% bovine serum albumin for 2 h at room temperature. After a second wash, PBS-diluted fecal or serum samples were added and incubated for 1h30 (fecal samples) or 2 h (serum) at room temperature. After washing, horseradish-peroxidase-conjugated goat anti-human IgA (1:2000 dilution) (Southern Biotech, Birmingham, AL, USA) or HRP-labeled Goat anti-Human IgG (1:7500 dilution) (Nordic MUbio, Susteren, The Netherlands) was added and incubated for 1h30 at room temperature. After a final wash, TMB substrate was added. After 20 min the reaction was stopped by adding 4 M H2SO4 and optical density (OD) was measured at 450 nm against a reference wavelength of 630 nm. For fecal samples, OD values were used as arbitrary units (AU) and corrected for fecal dry weight. For serum samples, a positive pool serum was used as calibration curve, and expressed as AU/mL.

Whole blood leucocyte subsets were quantified using an Automated cell counter. Serum C-reactive protein was measured using a high-sensitive commercial kit (Immundiagnostik AG, Bensheim, Germany).

Plasma IFN-γ, IL-1β, IL-6, IL-8 (CXCL8), TNF-α, IP-10 (CXCL10), and MIP-1α (CCL3), were measured using Luminex system (Bio-Rad).

### Ex vivo whole blood stimulation

Ex vivo whole blood stimulation was performed using TruCulture tubes (MyriadRBM, Austin TX, USA), preloaded with cell culture medium only (Null tubes), or supplemented with lipopolysaccharide (LPS tubes), according to manufacturer’s instructions with minor modifications. In short, 1 mL of heparinized whole blood was pipetted into each tube, and incubated in parallel in a dry heat block at 37 °C for 24 h. After incubation, the supernatant was separated from the cells, and the tubes were directly frozen and stored at − 80 °C. Supernatants were analyzed using the Mesoscale multiplex platform (Supplementary Table 1). Only cytokines/chemokines with median levels above 1 pg/mL were considered relevant for statistical analysis.

### Data and statistical analysis

Data are expressed as mean or median for continuous data or as frequency counts and percentages for categorical data. ELISA data were 10log-transformed to obtain parametric distribution. Comparisons of kinetic responses over time within and between subjects with standard dose (1E10 CFU) and high dose (5E10 CFU) inoculation, or low-responders and high-responders, were performed using one-way ANOVA (repeated measures if applicable) with Dunnett’s post hoc tests, or by two-way ANOVA with Bonferroni post hoc tests, respectively. For comparison of 2 data subsets, the change within subgroups at 2 timepoints was analyzed using the Wilcoxon Signed Rank test, and comparison between 2 subgroups at 1 timepoint was performed using the Wilcoxon rank sum test. A p-value < 0.05 was considered significant, and all tests were two-tailed. SAS version 9.4 and GraphPad Prism version 5.01 were used for statistical analysis.

High- and low-responder subsets were defined, based on a combination of clinical outcomes after the first *E. coli* challenge (percentage fecal wet weight, stool frequency, BSS, GIQLI total score, and GSRS domains diarrhea, indigestion and abdominal pain). Subjects were ranked, to identify the 12 highest and 12 lowest responders. High- and low-responders were equally distributed over the 1E10 and the 5E10 dose group (n = 6 subjects of each dose group within each responder group). Ex vivo whole blood stimulation was conducted in a subset of 6 high responders and 6 low responders (n = 3 subjects of each dose group within each responder group).

## Supplementary Information

Supplementary Information

## Abbreviations

BMI: Body mass index
BSS: Bristol Stool Score
CFA/II: Colonization factor antigen II
CFU: Colony-forming units
CRP: C-reactive protein
*E. coli*: *Escherichia coli*
ETEC: Enterotoxigenic *Escherichia coli*
GSRS: Gastro-intestinal Symptom Rating Scale
GIQLI: Gastro-intestinal Quality of Life Index
ICP-AES: Inductively coupled plasma-atomic emission spectrophotometry
LPS: Lipopolysaccharide
PCR: Polymerase chain reaction
