# Supplementary material for: Characterization of the pathophysiological determinants of diarrheagenic Escherichia coli infection using a challenge model in healthy adults
Source: Sci Rep. 2021 Mar 15;11:6060. doi: 10.1038/s41598-021-85161-1 (PMC7960709; doi:10.1038/s41598-021-85161-1)
Supplement: Supplementary file 1 — Supplementary Information [file 41598_2021_85161_MOESM1_ESM.pdf]

# Characterization of the pathophysiological determinants of diarrheagenic *Escherichia coli* infection using a challenge model in healthy adults.

**Running title: Human *E. coli* infection challenge model**

**Els Van Hoffen,[1] Annick Mercenier,[2,5*] Karine Vidal,[2*] Jalil Benyacoub,[2] Joyce Schloesser,[1] Alwine Kardinaal,[1] Elly Lucas-van de Bos,[1] Ingrid van Alen,[1] Iris Roggero,[2] Kim Duintjer,[1,3] Anneke Berendts,[1] Ruud Albers,[4] Michiel Kleerebezem,[3] Sandra Ten Bruggencate[1]**

[1] Department of Nutrition & Health, NIZO, Ede, the Netherlands

[2] Nestlé Institute of Health Sciences, Gastrointestinal Health, Nestlé Research, Lausanne, Switzerland

[3] Host Microbe Interactomics Group, Department of Animal Sciences, Wageningen University and Research, Wageningen, the Netherlands

[4] NutriLeads, Wageningen, The Netherlands ([5] Current affiliation)

* These authors contributed equally to this manuscript.

**Corresponding author:**

Joyce Schloesser, MSc

Department of Nutrition & Health, NIZO

PO Box 20, 6710 BA Ede, The Netherlands

Phone: +31 318 659 511

E-mail: Joyce.Schloesser@nizo.com

## SUPPLEMENTARY FIGURES

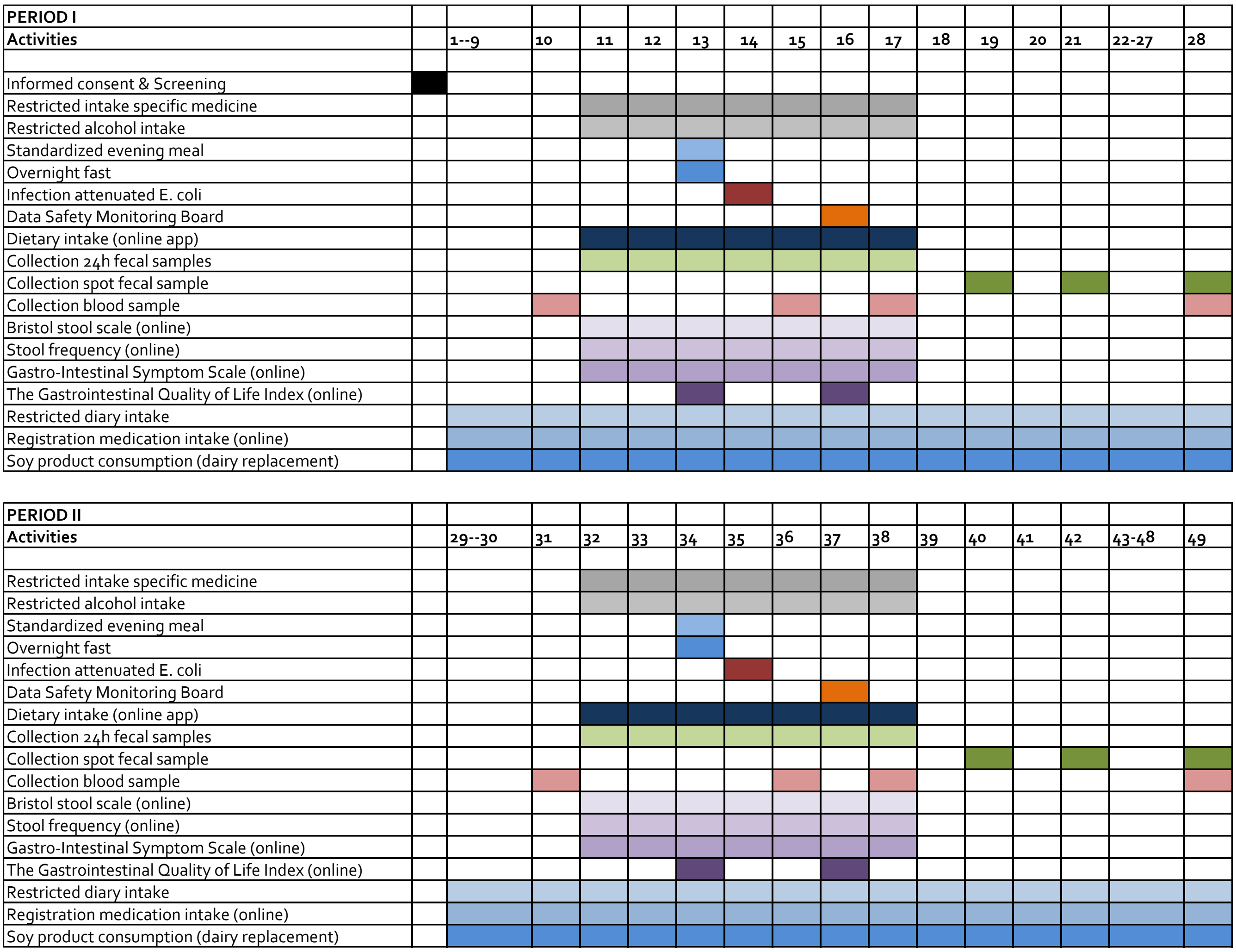


**Supplementary Figure 1:** Study schedule outlining all study guidelines, questionnaires and biological sampling before and after the first (day 14) and second (day 35) *E. coli* E1392/75-2A inoculation.

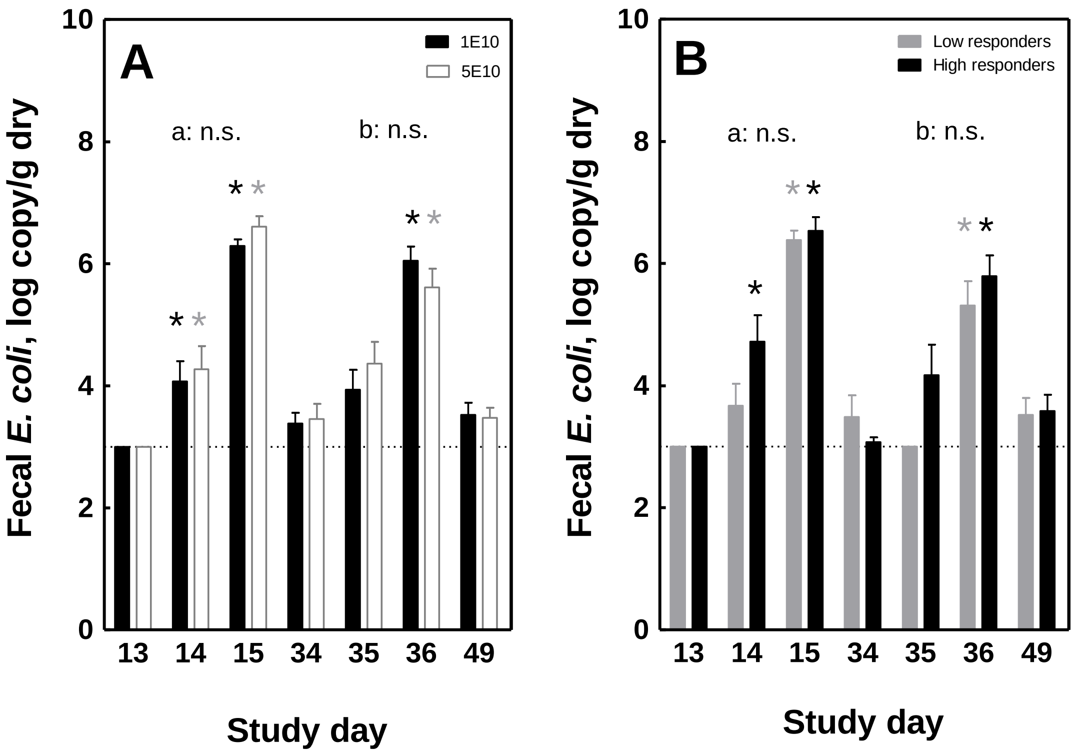


**Supplementary Figure 2. Fecal *E.coli* excretion in fecal samples in subjects before and after the first (day 14) and second (day 35) *E.coli* E1392/75-2A challenge.** Fecal *E.coli* E1392/75-2A was detected in fecal samples by qPCR, using Colonization Surface antigen 3 (CS3)-specific primers. Results are shown as means ± SEM for the dose groups (**A**; 1E10 CFU n=20, black bars; 5E10 CFU n=22, open bars) and for the high- and low-responders (**B**; high-responders n=12, black bars; low-responders n=12, grey bars). Dotted line indicates the limit of detection (LLD). Samples below LLD were given the LLD value of 1E3. Timepoint at which significance is reached as compared to baseline day 13 (within the first infection period) or day 34 (within the second infection period) is indicated by *, with $p<0.05$ within the specified group (Kruskal-Wallis test; **A**: black *: within standard dose 1E10 CFU; grey *: within high dose 5E10 CFU; **B**: black *: within high-responders; grey *: within low-responders). Significant differences in the overall response between standard and high dose, or between high- and low-responders (two-way ANOVA) are indicated separately for the first (a, days 13-34) and second (b, days 34-49) infection period; n.s., not significant.

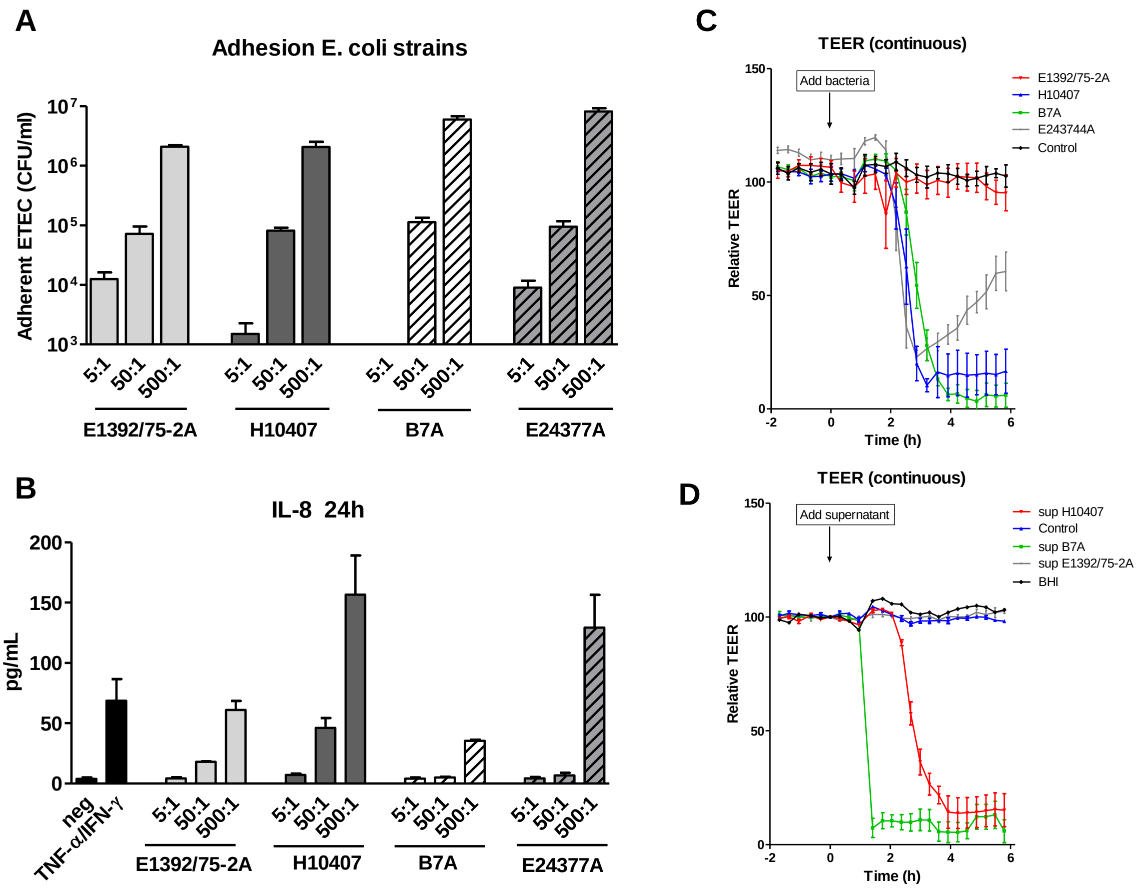


**Supplementary Figure 3:** ***In vitro*** **characteristics of** ***E. coli*** **E1392/75-2A**

**A, B.** Adhesion to and production of IL-8 by confluent Caco-2 cells was compared between *E. coli* strain E1392/75-2A and 3 wild-type strains: *E. coli* H10407, B7A, and E243744A. Caco-2 cells were incubated for 1 h at 37°C with the various strain in triplicate at a multiplicity of infection (MOI) of 5:1, 50:1 and 500:1, in antibiotics-free medium. For adhesion (**A**), cells were washed, and lysed in sterile water. *E. coli* adhesion was measured by plating the lysates on agar. For IL-8 production (**B**), monolayers were washed and further incubated with culture medium containing 50 µg/ml gentamicin and 1% penicillin/streptomycin. Supernatants were collected after 24 h, centrifuged to remove cell debris and stored at -40°C. The concentration of IL-8 was determined using the PeliKine Compact human IL-8 ELISA (Sanquin, Amsterdam, The Netherlands). Results are shown as mean ± sem of triplicate measurements. For statistical analysis of adhesion and IL-8 production, all data were compared to *E.coli* E1392/75-2A and analyzed by Kruskal Wallis test. No significant differences were observed.

**C, D.** The effect of *E. coli* E1392/75-2A and 3 toxin-producing *E. coli* strains H10407, B7A, and E243744A on epithelial barrier function was analyzed using differentiated Caco-2 cells in a transwell model. To continuously measure TEER over time, a CellZscope (Nanoanalytics GmbH, Münster, Germany) was used. Caco-2 cells ($2*10^4$ cells/well) were seeded in transparent 24-well filter (0.4 µm) inserts (Falcon BD Biosciences, San Jose, CA, USA) and cultured for 2 weeks, reaching a typical transepithelial electrical resistance of 400 Ohm*cm$^2$. The plates were placed in the CellZscope at least one day prior to the experiment to allow for equilibration. In the basolateral compartment, 800 µL and in the apical compartment 450 µL of culture medium (without antibiotics) was added, respectively. At the start of the actual experiment (t=0 h), 50 µL of bacterial suspension or culture medium was added to the apical compartment. The bacterial suspension was added at an MOI of 10:1 (**C**). Bacterial spent culture supernatant (**D**) was harvested after overnight growth at 37°C in BHI medium, followed by removal of the bacteria by centrifugation and passing the supernatant through a 0.2 µm filter (Whatman). Caco-2 cells were exposed to these spent culture supernatants (5% v/v), or to the same amount of BHI medium or PBS (**D**). TEER readout at the time of addition of bacteria or bacterial spent media was set at 100%, and all results presented are the mean ± SEM of triplicate measurements.

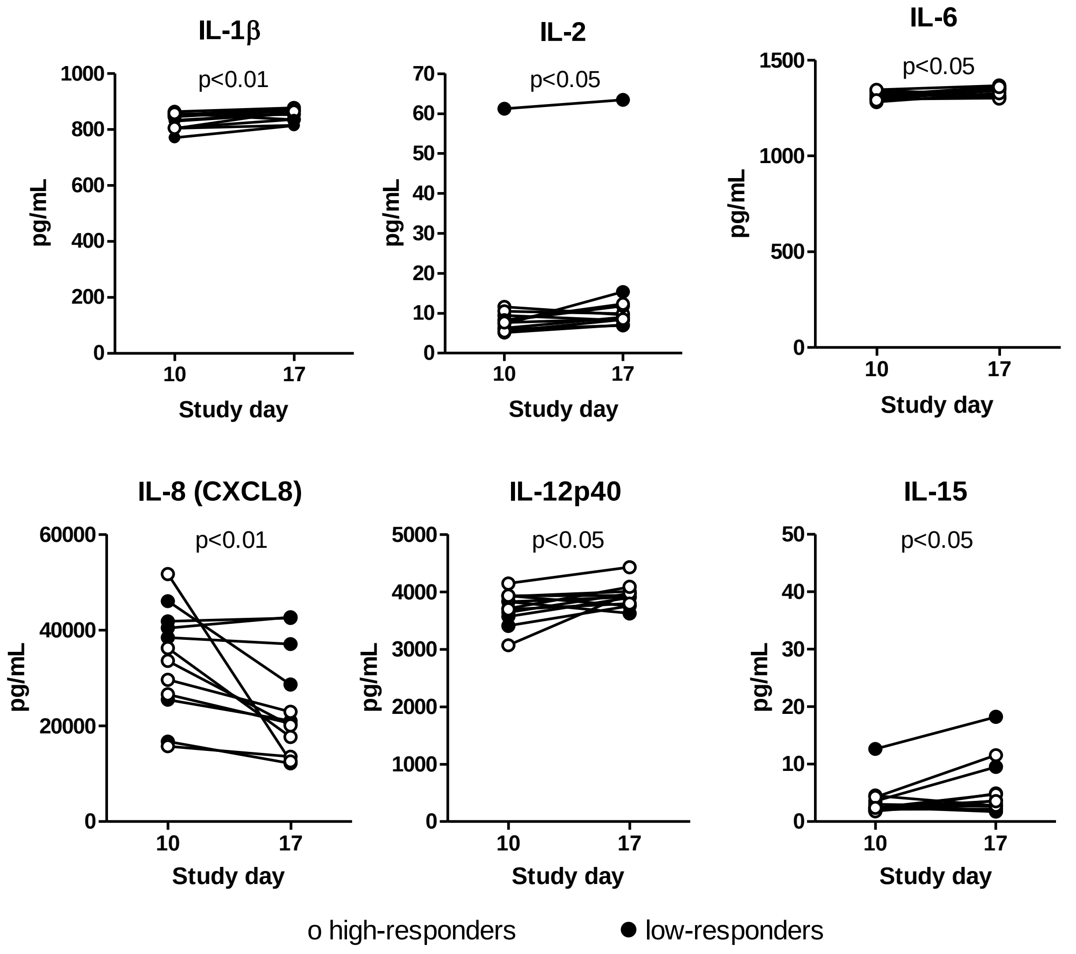


**Supplementary Figure 4. *Ex vivo* cytokine and chemokine production in LPS-stimulated whole blood before and after *E. coli* challenge.** *Ex vivo* stimulation of whole blood cells was performed in a subset of subjects (n=6 low-responders, closed circles, and n=6 high-responders, open circles). Subjects were orally infected with a live attenuated *E. coli* strain E1392/75-2A on study day 14. Whole blood stimulation was performed on study day 10 and 17, using LPS TruCulture tubes (medium containing LPS). No difference was observed between low- and high-responders, therefore the data of both subgroups were combined in statistical analysis. From the total panel of cytokines and chemokines that were analysed, the figures are shown for those that changed significantly after challenge (Wilcoxon Signed Rank test).

**Supplementary Table 1A. Overview of V-plex cytokine and chemokine data of TruCulture null-stimulated whole blood.**

| | Day 10 (before *E. coli* infection) | | Day 17 (3 days after *E. coli* infection) | |
|---|---|---|---|---|
| | Median | Range | Median | Range |
| **Cytokine panel 1** | | | | |
| GM-CSF | 0.09 | 0.05-1.03 | 0.10 | 0.07-0.84 |
| IL-1α | 0.92 | 0.38-4.05 | 0.50 | 0.07-14.0 |
| IL-5 | 0.14 | 0.06-2.09 | 0.17 | 0.08-2.13 |
| IL-7 | 4.00 | 2.64-5.69 | 4.07 | 2.88-4.94 |
| IL-12p40 | 3.38 | 2.18-8.19 | 4.68 | 2.48-157 |
| IL-15 | 0.56 | 0.34-1.09 | 0.63 | 0.51-1.25 |
| IL-16 | 28.14 | 11.4-35.8 | 25.63 | 8.49-34.1 |
| IL-17 | 0.07 | 0.02-0.20 | 0.18 | 0.06-2.23 |
| TNF-β | 0.12 | 0.07-2.58 | 0.16 | 0.05-2.49 |
| VEGF | 18.58 | 6.82-31.5 | 18.31 | 2.18-68.7 |
| **Chemokine panel 1** | | | | |
| Eotaxin (CCL11) | 341.70 | 180-427 | 311.30 | 162-481 |
| Eotaxin-3 (CCL26) | 96.83 | 54.7-288 | 123.60 | 48.1-324 |
| IL-8 (CXCL8) | 602.50 | 79.7-3431 | 685.30 | 191-4728 |
| IP-10 (CXCL10) | 39.19 | 25.4-117 | 72.29 | 29.6-2834 |
| MCP-1 (CCL2) | 46.12 | 28.8-91.3 | 41.63 | 37.0-1290 |
| MCP-4 (CCL13) | 25.60 | 12.5-57.1 | 25.74 | 17.8-58.4 |
| MDC (CCL22) | 242.40 | 129-444 | 295.00 | 133-487 |
| MIP-1α (CCL3) | 10.93 | 5.41-34.1 | 24.08 | 8.87-2449 |
| MIP-1β (CCL4) | 76.31 | 29.4-115 | 165.00 | 42.7-1082 |
| TARC (CCL17) | 2.21 | 1.05-6.17 | 2.41 | 1.11-6.46 |
| **Proinflammatory panel 1** | | | | |
| IFN-γ | 1.98 | 0.94-5.76 | 3.75 | 1.79-71.31 |
| IL-1β | 0.01 | 0.00-0.80 | 0.18 | 0.00-58.8 |
| IL-2 | 0.08 | 0.00-2.58 | 0.47 | 0.10-3.99 |
| IL-4 | 0.07 | 0.00-0.17 | 0.07 | 0.03-1.18 |
| IL-6 | 0.69 | 0.28-1.51 | 1.08 | 0.39-1140 |
| IL-8 (CXCL8) | 34.39 | 12.8-108 | 59.01 | 15.6-2705 |
| IL-10 | 0.10 | 0.02-0.84 | 0.24 | 0.04-1.90 |
| IL-12p70 | 0.12 | 0.05-2.39 | 0.29 | 0.00-6.58 |
| IL-13 | 2.21 | 0.45-15.8 | 4.22 | 0.53-16.1 |
| TNF-α | 2.14 | 1.06-4.08 | 3.28 | 1.37-728 |

* All from Meso Scale Diagnostics, Rockville, MD, USA
Green: analyzed; (near) significantly increased after the first *E. coli* infection
Orange: analyzed; not significantly changed.
White: median levels <1 pg/mL; not included in statistical analysis.

**Supplementary Table 1B. Overview of V-plex cytokine and chemokine data – LPS stimulation**

| | **Day 10 (before *E. coli* infection)** | | **Day 17 (3 days after *E. coli* infection)** | |
|---|---|---|---|---|
| | Median | Range | Median | Range |
| **Cytokine panel 1*** | | | | |
| GM-CSF | 13 | 4-61 | 12 | 5-31 |
| IL-1α | 1755 | 100-3976 | 1825 | 57-3916 |
| IL-5 | 2 | 1-18 | 2 | 0-15 |
| IL-7 | 9 | 5-26 | 11 | 5-25 |
| IL-12p40 | 3700 | 1926-4145 | 3794 | 1624-4427 |
| IL-15 | 3 | 1-13 | 3 | 1-18 |
| IL-16 | 41 | 14-83 | 43 | 16-92 |
| IL-17 | 4 | 1-11 | 4 | 1-7 |
| TNF-β | 3 | 0-29 | 4 | 0-26 |
| VEGF | 3 | 1-40 | 4 | 2-35 |
| **Chemokine panel 1*** | | | | |
| Eotaxin (CCL11) | 331 | 163-728 | 316 | 163-810 |
| Eotaxin-3 (CCL26) | 331 | 282-650 | 365 | 264-619 |
| IL-8 (CXCL8) | 32601 | 9908-57559 | 22932 | 12196-58143 |
| IP-10 (CXCL10) | 4951 | 865-46851 | 1547 | 579-5727 |
| MCP-1 (CCL2) | 1223 | 795-1332 | 1193 | 678-1342 |
| MCP-4 (CCL13) | 47 | 30-81 | 42 | 28-80 |
| MDC (CCL22) | 979 | 464-2549 | 789 | 391-2446 |
| MIP-1α (CCL3) | 2920 | 2704-3110 | 2816 | 2703-3133 |
| MIP-1β (CCL4) | 1068 | 1002-1103 | 1070 | 980-1123 |
| TARC (CCL17) | 4 | 2-9 | 4 | 2-8 |
| **Proinflammatory panel 1*** | | | | |
| IFN-γ | 8069 | 1508-11251 | 7018 | 204-10962 |
| IL-1β | 839 | 771-889 | 854 | 794-878 |
| IL-2 | 8 | 5-61 | 10 | 6-63 |
| IL-4 | 7 | 4-12 | 7 | 4-23 |
| IL-6 | 1306 | 1261-1353 | 1335 | 1270-1368 |
| IL-8 (CXCL8) | 3161 | 2971-3268 | 3102 | 2976-3253 |
| IL-10 | 81 | 21-158 | 60 | 20-162 |
| IL-12p70 | 40 | 28-123 | 43 | 19-123 |
| IL-13 | 24 | 21-66 | 25 | 19-66 |
| TNF-α | 3729 | 3542-3865 | 3711 | 3507-3911 |

* All from Meso Scale Diagnostics, Rockville, MD, USA
Green: analyzed; (near) significantly increased after the first *E. coli* infection
Orange: analyzed; not significantly changed.
